# A Weighted Belief Entropy-Based Uncertainty Measure for Multi-Sensor Data Fusion

**DOI:** 10.3390/s17040928

**Published:** 2017-04-22

**Authors:** Yongchuan Tang, Deyun Zhou, Shuai Xu, Zichang He

**Affiliations:** School of Electronics and Information, Northwestern Polytechnical University, Xi’an 710072, China; dyzhou@nwpu.edu.cn (D.Z.); xushuainwpu@mail.nwpu.edu.cn (S.X.); hezichang@mail.nwpu.edu.cn (Z.H.)

**Keywords:** uncertainty measure, Dempster–Shafer evidence theory, Deng entropy, weighted belief entropy, sensor data fusion

## Abstract

In real applications, how to measure the uncertain degree of sensor reports before applying sensor data fusion is a big challenge. In this paper, in the frame of Dempster–Shafer evidence theory, a weighted belief entropy based on Deng entropy is proposed to quantify the uncertainty of uncertain information. The weight of the proposed belief entropy is based on the relative scale of a proposition with regard to the frame of discernment (FOD). Compared with some other uncertainty measures in Dempster–Shafer framework, the new measure focuses on the uncertain information represented by not only the mass function, but also the scale of the FOD, which means less information loss in information processing. After that, a new multi-sensor data fusion approach based on the weighted belief entropy is proposed. The rationality and superiority of the new multi-sensor data fusion method is verified according to an experiment on artificial data and an application on fault diagnosis of a motor rotor.

## 1. Introduction

In the age of artificial intelligence, sensors play quite an important role for environment sensing and information acquisition. At the same time, sensors may be affected by the complicated application environment. Thus, multi-sensor modeling and sensor data fusion are important issues in many real applications [1,2,3,4,5,6,7]. Driven by real applications, many methods have been proposed for multi-sensor modeling and sensor data fusion [8], including neural network models [1,9], belief function theory [10,11], Dempster–Shafer evidence theory [12,13,14], fuzzy set theory [15], Z-Numbers [16], and so on [17]. Furthermore, accompanied by multi-sensor data fusion, how to measure the uncertain degree or the reliability of sensor reports with heterogeneous sources is still an open issue. This paper focuses on multi-sensor data fusion by firstly proposing a new uncertainty measure and then designing a new uncertainty measure-based sensor data fusion approach.

Dempster–Shafer evidence theory [18,19] is effective in uncertain information modeling and processing, and it has been widely used in many fields, such as multiple attribute decision making [20,21,22], risk analysis [23,24,25,26,27,28], pattern recognition [29,30,31,32,33,34], fault diagnosis [11,12,13], controller design [35,36] and so on [37,38,39]. Although Dempster–Shafer evidence theory is an effective method for information processing, the classical Dempster’s rule of combination can’t be used directly for conflict sensor data fusion, especially when there exists highly conflicting data, which may lead to counterintuitive results [40,41]. One way to handle the conflict information in real applications, such as sensor data fusion, is to quantify the uncertainty before applying data fusion [11,13,42]. However, how to measure the uncertainty of uncertain information in the framework of Dempster–Shafer evidence theory is still an open issue [43,44,45].

Shannon entropy is an admitted way for measuring information volume [46], which is a typical way for uncertainty measure in the probabilistic framework. Although Shannon entropy has been generalized to many fields, for example, as a generalization of Shannon entropy, network entropy is an effective measurement for measuring the complexity of networks [47,48,49,50], and Shannon entropy can’t be used directly among mass functions in the framework of Dempster–Shafer evidence theory because a mass function is a generalized probability assigned on the power set of the frame of discernment (FOD). To address this issue, many uncertainty measures in Dempster–Shafer framework are proposed, such as Hohle’s confusion measure [51], Yager’s dissonance measure [52], the weighted Hartley entropy [53], Klir and Ramer’s discord measure [54], Klir and Parviz’s strife measure [55] and George and Pal’s conflict measure [56] and so on [43,44]. However, the existed methods may not be that effective in some cases [57]. Recently, another uncertainty measure named Deng entropy is proposed [57]. Although Deng entropy had been successfully applied in some real applications [11,12,13,14,16], Deng entropy didn’t take into consideration of the scale of FOD, which means a loss of available and valuable information in information processing.

In order to overcome this shortage of Deng entropy, a weighted belief entropy based on Deng entropy is proposed in this paper. The weighted belief entropy takes advantage of the relative scale of a proposition with respect to the FOD. In other words, the cardinality of the proposition and the number of element in FOD are used to define the weight factor in the proposed uncertain measure. After that, based on the proposed measure, a new sensor data fusion approach is proposed. In the proposed method, the weighted belief entropy is used to preprocess the conflict data by measuring the uncertain degree of each body of evidence (BOE). Finally, the conflict data can be fused by Dempster’s rule of combination.The effectiveness of the weighted belief entropy is verified with the numerical example in [57]. In addition, the new sensor data fusion method is applied on fault diagnosis of a motor rotor to show the capacity of the new method in real application.

The rest of this paper is organized as follows. In Section 2, the preliminaries on Dempster–Shafer evidence theory, Shannon entropy, Deng entropy and some uncertainty measures in Dempster–Shafer framework are briefly introduced. In Section 3, the weighted belief entropy is proposed. In Section 4, a new sensor data fusion approach based on the weighted belief entropy is proposed. In Section 5, a numerical example for the new method is presented. In Section 6, the proposed sensor data fusion method is used for fault diagnosis of a motor rotor. The conclusions are given in Section 7.

## 2. Preliminaries

Some preliminaries are briefly introduced in this section, including Dempster–Shafer evidence theory [18,19], Shannon entropy [46], Deng entropy [57] and some other typical uncertainty measures in Dempster–Shafer framework [51,52,53,54,55,56].

### 2.1. Dempster–Shafer Evidence Theory

Let $\Omega \;=\left\{\theta_{1},\theta_{2},\ldots ,\theta_{i},\ldots ,\theta_{N}\right\}$ be a finite nonempty set of mutually exclusive and exhaustive events, Ω is called the *frame of discernment* (FOD). The power set of Ω, denoted as $2^{\Omega }$, is composed of $2^{N}$ elements denoted as follows:

(1) $$2^{\Omega }=\left\{\emptyset ,\left\{\theta_{1}\right\},\left\{\theta_{2}\right\},\ldots ,\left\{\theta_{N}\right\},\left\{\theta_{1},\theta_{2}\right\},\ldots ,\left\{\theta_{1},\theta_{2},\ldots ,\theta_{i}\right\},\ldots ,\Omega \right\}.$$

A *mass function* *m* is defined as a mapping from the power set $2^{\Omega }$ to the interval [0,1], which satisfies the following conditions [18,19]:

(2) $$m\left(\emptyset \right)=0,\sum_{A\in \Omega }m\left(A\right)=1.$$

If $m\left(A\right)>0$, then *A* is called a *focal element*, the mass function $m\left(A\right)$ represents how strongly the evidence supports the proposition *A*.

A *body of evidence* (BOE), also known as a *basic probability assignment* (BPA) or *basic belief assignment* (BBA), is represented by the focal sets and their associated mass value:

(3) $$\left(ℜ,m\right)=\left\{\langle A,m\left(A\right)\rangle :A\in 2^{\Omega },m\left(A\right)>0\right\},$$

where *ℜ* is a subset of the power set $2^{\Omega }$, and each A∈ℜ has an associated nonzero mass value $m\left(A\right)$.

A BPA *m* can also be represented by its associate belief function Bel and plausibility function Pl respectively, defined as follows:

(4) $$Bel\left(A\right)=\sum_{\phi \neq B\subseteq A}m\left(B\right)\;and\;Pl\left(A\right)=\sum_{B\cap A\neq \phi }m\left(B\right).$$

In Dempster–Shafer evidence theory, two independent mass functions, denoted as $m_{1}$ and $m_{2}$, can be combined with Dempster’s rule of combination defined as [18,19]:

(5) $$m\left(A\right)=\left(m_{1}⊕m_{2}\right)\left(A\right)\;=\frac{1}{1-k}\sum_{B\cap C=A}m_{1}\left(B\right)m_{2}\left(C\right),$$

where *k* is a normalization constant representing the *degree of conflict* between $m_{1}$ and $m_{2}$, *k* is defined as [18,19]:

(6) $$k=\sum_{B\cap C=\emptyset }m_{1}\left(B\right)m_{2}\left(C\right).$$

### 2.2. Shannon Entropy

As an uncertainty measure of information volume in a system or process, Shannon entropy plays a central role in information theory. Shannon entropy indicates that the information volume of each piece of information is directly connected to its uncertain degree.

Shannon entropy, as the information entropy, is defined as follows [46]:

(7) $$H=-\sum_{i=1}^{N}p_{i}\log_{b}p_{i},$$

where *N* is the number of basic states, $p_{i}$ is the probability of state *i*, and $p_{i}$ satisfies $\sum_{i=1}^{N}p_{i}=1$. If the unit of information is bits, then b=2.

### 2.3. Deng Entropy

Deng entropy is a generalization of Shannon entropy in Dempster–Shafer framework. If the information is modelled in the framework of a probability theory, Deng entropy can be degenerated to Shannon entropy. Deng entropy, denoted as $E_{d}$, is defined as follows [57]:

(8) $$E_{d}\left(m\right)=-\sum_{A\subseteq X}m\left(A\right)\log_{2}\frac{m\left(A\right)}{2^{\left|A\right|}-1},$$

where $\left|A\right|$ denotes the cardinality of the proposition *A*, and *X* is the FOD. If and only if the mass value is assigned to single elements, Deng entropy can be degenerated to Shannon entropy, in this case, the form of Deng entropy is as follows:

(9) $$E_{d}\left(m\right)=-\sum_{A\subseteq X}m\left(A\right)\log_{2}\frac{m\left(A\right)}{2^{\left|A\right|}-1}=-\sum_{A\subseteq X}m\left(A\right)\log_{2}m\left(A\right).$$

For more details about Deng entropy, please refer to [57].

### 2.4. Uncertainty Measures in Dempster–Shafer Framework

In this section, some other typical uncertainty measures in the framework of Dempster–Shafer evidence theory are briefly introduced. Assume that *X* is the FOD, *A* and *B* are focal elements of the mass function, and $\left|A\right|$ denotes the cardinality of *A*. Then, the definitions of different uncertainty measures are shown as follows.

#### 2.4.1. Hohle’s Confusion Measure

Hohle’s confusion measure, denoted as $C_{H}$, is defined as follows [51]:

(10) $$C_{H}\left(m\right)=-\sum_{A\subseteq X}m\left(A\right)\log_{2}Bel\left(A\right).$$

#### 2.4.2. Yager’s Dissonance Measure

Yager’s dissonance measure, denoted as $E_{Y}$, is defined as follows [52]:

(11) $$E_{Y}\left(m\right)=-\sum_{A\subseteq X}m\left(A\right)\log_{2}Pl\left(A\right).$$

#### 2.4.3. Dubois and Prade’s Weighted Hartley Entropy

Dubois and Prade’s weighted Hartley entropy, denoted as $E_{DP}$, is defined as follows [53]:

(12) $$E_{DP}\left(m\right)=\sum_{A\subseteq X}m\left(A\right)\log_{2}\left|A\right|.$$

#### 2.4.4. Klir and Ramer’s Discord Measure

Klir and Ramer’s discord measure, denoted as $D_{KR}$, is defined as follows [54]:

(13) $$D_{KR}\left(m\right)=-\sum_{A\subseteq X}m\left(A\right)\log_{2}\sum_{B\subseteq X}m\left(B\right)\frac{\left|A\cap B\right|}{\left|B\right|}.$$

#### 2.4.5. Klir and Parviz’s Strife Measure

Klir and Parviz’s strife measure, denoted as $S_{KP}$, is defined as follows [55]:

(14) $$S_{KP}\left(m\right)=-\sum_{A\subseteq X}m\left(A\right)\log_{2}\sum_{B\subseteq X}m\left(B\right)\frac{\left|A\cap B\right|}{\left|A\right|}.$$

#### 2.4.6. George and Pal’s Conflict Measure

The total conflict measure proposed by George and Pal, denoted as $TC_{GP}$, is defined as follows [56]:

(15) $$TC_{GP}\left(m\right)=\sum_{A\subseteq X}m\left(A\right)\sum_{B\subseteq X}m\left(B\right)\left(1-\frac{\left|A\cap B\right|}{\left|A\cup B\right|}\right).$$

## 3. The Proposed Uncertainty Measurement

In this section, a weighted belief entropy based on Deng entropy is proposed. In the framework of Dempster–Shafer evidence theory, the uncertain information is represented not only by mass functions, the FOD is also a source of uncertainty, for example, the number of elements in a FOD can be changed even if the mass value of each proposition keeps still. However, the existed belief entropy, such as Dubois and Prade’s weighted Hartley entropy and Deng entropy, only takes into consideration of mass functions, the cardinality of the proposition and the scale of FOD are ignored. This may lead to information loss in information processing.

### 3.1. Weighted Belief Entropy

By addressing more available information in the evidence, includes the scale of FOD, denoted as $\left|X\right|$, and the relative scale of a focal element with respect to the FOD, denoted as $\left(\left|A\right|/\left|X\right|\right)$. The new belief entropy named the weighted belief entropy is proposed as follows:

(16) $$E_{Wd}\left(m\right)=-\sum_{A\subseteq X}\frac{\left|A\right|m\left(A\right)}{\left|X\right|}\log_{2}\frac{m\left(A\right)}{2^{\left|A\right|}-1},$$

where *X* is the FOD, *A* is the focal element of the mass function, $\left|A\right|$ denotes the cardinality of the proposition *A* and $\left|X\right|$ is the number of elements in FOD.

Compared with Deng entropy, the weighted belief entropy addresses more uncertain information in BOE, which can contribute to a more accurate information processing procedure in real applications. In the next subsection, a numerical example is used to show the effectiveness of the new measure, as well as making a comparison with some other typical uncertainty measures in Dempster–Shafer framework.

### 3.2. Numerical Example

In order to test the capacity and superiority of the weighted belief entropy, recall the example in [57].

Consider the mass function $m\left(\left\{6\right\}\right)=0.05$, $m\left(\left\{3,4,5\right\}\right)=0.05$, $m\left(T\right)=0.8$ and $m\left(X\right)=0.1$ in a FOD $X=\left\{1,2,...,14,15\right\}$ with fifteen elements denoted as Element 1, ..., and Element 15. *T* represents a variable subset with its number of element changes from Element 1 to Element 14, as is shown in Table 1.

Deng entropy $E_{d}$ in Equation (8) and the weighted belief entropy $E_{Wd}$ in Equation (16) are calculated with a changed proposition, and the results are shown in Table 1. According to Table 1, the values of weighted belief entropy are all smaller than that of Deng entropy. This is reasonable because more information in the BOE is taken into consideration with the weighted belief entropy, which means the weighted belief entropy has less information loss than Deng entropy. By reducing the uncertain degree, the new measure can be more accurate than Deng entropy for uncertainty measure in information processing.

**Figure 1:**
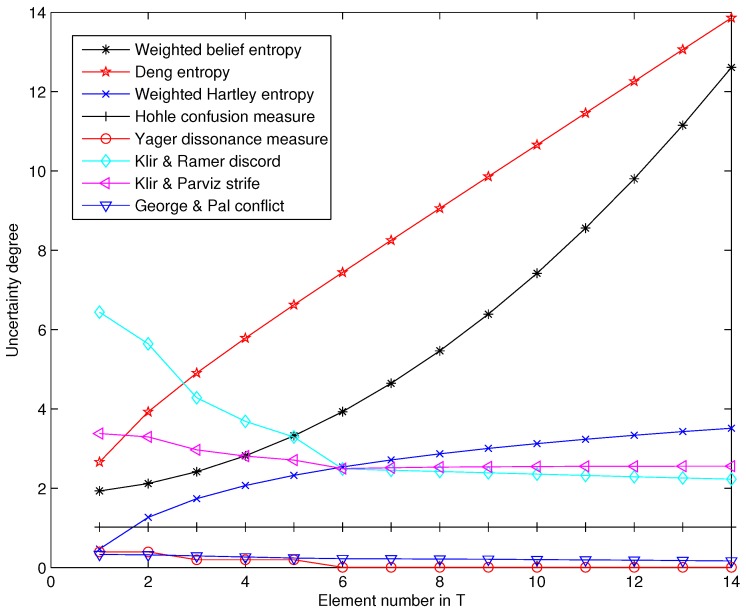
Comparison between the weighted belief entropy and other uncertainty measures.

Figure 1 shows the comparison results of different uncertainty measures in Dempster–Shafer framework. The uncertain degree measured by Hohle’s confusion measure never changes with the variation of the element number in proposition *T*, thus it cannot measure the variance of uncertainty in this case. Similar to the confusion measure, Yager’s dissonance measure has a limited capacity of uncertainty measure in this case. The uncertain degree measured by Klir and Ramer’s discord measure, Klir and Parviz’s strife measure and George and Pal’s conflict measure all decreases with the increase of the element number in proposition *T*. Thus, the confusion measure, dissonance measure, discord measure, strife measure and conflict measure can’t effectively measure the increase of uncertain degree in BOE in this case. With Dubois and Prade’s weighted Hartley entropy, Deng entropy and the weighted belief entropy, the uncertain degree increases significantly with the increase of the element number in proposition *T*. However, the weighted Hartley entropy and Deng entropy have significant information loss compared with the weighted belief entropy because the uncertain information modelled as the scale of FOD ($\left|X\right|$) hasn’t been addressed in Equation (8) and Equation (12). Above all, the weighted belief entropy is more reasonable than the other uncertain measures in this case.

## 4. The Weighted Belief Entropy-Based Sensor Data Fusion Approach

**Figure 2:**
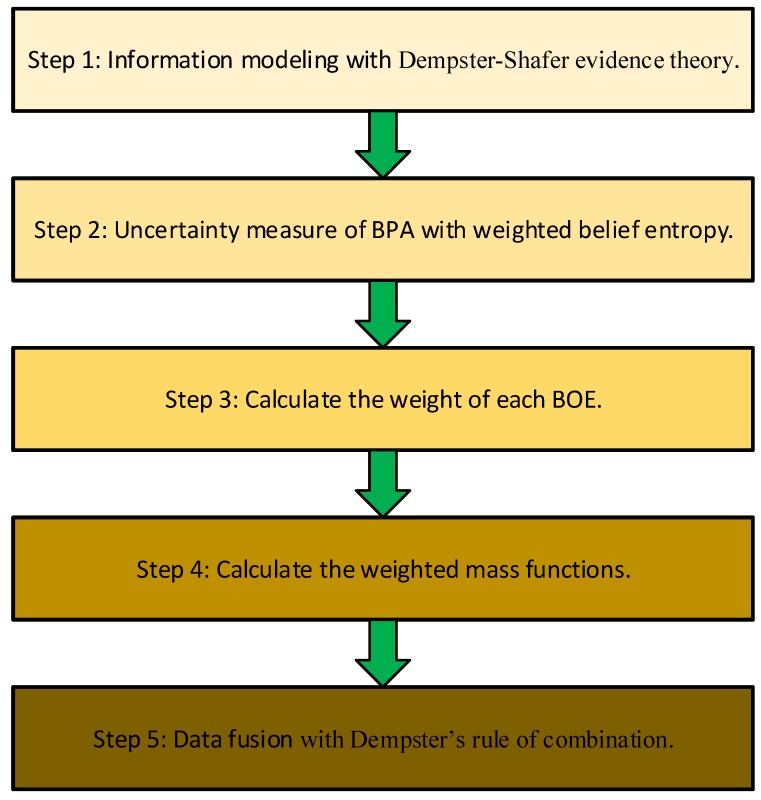
The flow chart of sensor data fusion based on the weighted belief entropy.

In order to fuse conflict sensor data properly in real applications, in this section, a multi-sensor data fusion approach is proposed based on the new measure. In the proposed method, the uncertain degree of evidence is measured by the new weighted belief entropy. Five steps are included in the proposed multi-sensor data fusion method, as is shown in Figure 2.

The details of the five steps in Figure 2 are presented as follows.

Step 1 Uncertain data modeling with BPA.
  In real applications, the information or data can be any style, so the first step of information processing in the frame of the Dempster–Shafer evidence theory mainly focuses on modeling uncertain information with BPAs in BOE.
Step 2 Uncertainty measure of BPA with weighted belief entropy.
  The uncertain degree of information modeled by BPAs in the 1st step needs to be measured with a proper uncertainty measure before further processing. In the proposed method, the weighted belief entropy is used to measure the uncertain degree of each BOE.
  For the *i*th BOE (i=1,2,⋯,n), its corresponding uncertain degree with the weighted belief entropy $E_{Wd}$ is calculated as follows:
  (17) $$E_{Wd}\left(m_{i}\right)=-\sum_{A\subseteq X}\frac{\left|A\right|m_{i}\left(A\right)}{\left|X\right|}\log_{2}\frac{m_{i}\left(A\right)}{2^{\left|A\right|}-1}.$$
Step 3 Calculate the weight of each BOE.
  Based on the value of weighted belief entropy, the weight of each BOE can be calculated. Generally, the weight of each BOE has a positive correlation with the uncertainty measure of each BPA [12].
  For the *i*th BOE (i=1,2,⋯,n), its weight based on the uncertain degree measured by the weighted belief entropy is calculated as follows:
  (18) $$w_{i}=\frac{E_{Wd}\left(m_{i}\right)}{\sum_{i=1}^{n}E_{Wd}\left(m_{i}\right)}.$$
Step 4 Calculate the weighted mass functions.
  The weighted mass function of each proposition is calculated for the final data fusion.
  For each proposition *A* in the BOE, the weighted mass function can be calculated as follows:
  (19) $$m_{w}\left(A\right)=\sum_{i=1}^{n}w_{i}m_{i}\left(A\right).$$
Step 5 Data fusion with Dempster’s rule of combination.
  In the proposed method, the conflict among different evidence is transformed and measured by the weighted belief entropy, and now data fusion can be completed with Dempster’s rule of combination.
  For each proposition *A* in the BOE, the combination result can be obtained by calculating Dempster’s rule of combination with (n-1) times:
  (20) $$m\left(A\right)={\left({\left({\left({\left(m_{w}⊕m_{w}\right)}_{1}⊕m_{w}\right)}_{2}...⊕m_{w}\right)}_{\left(n-2\right)}⊕m_{w}\right)}_{\left(n-1\right)}\left(A\right),n\geq 2.$$

## 5. Experiment with Artificial Data

In order to verify the rationality and effectiveness of the proposed multi-sensor data fusion method, an experiment is performed in this section. The experiment in [58] is recalled for the convenience of making a comparison with some other methods.

Considering a target recognition problem, three potential targets are denoted as *A*, *B* and *C*, respectively. The evidence reported by five sensors is modeled as BPAs, as is shown in Table 2. Intuitively, as is described in [58], the report from the 2nd sensor is contrary to the other four sensors and *A* will be the recognized target with the highest belief.

Execute the method in Figure 2 of Section 4. The result of the 1st step is adopted from [58], and the BPAs are presented in Table 2.

For the 2nd step, with Equation (17), the weighted belief entropy of each sensor report is calculated as follows:

$$E_{Wd}\left(m_{1}\right)=-\sum_{A\subseteq X}\frac{\left|A\right|m_{1}\left(A\right)}{\left|X\right|}\log_{2}\frac{m_{1}\left(A\right)}{2^{\left|A\right|}-1}=0.5221,$$

$$E_{Wd}\left(m_{2}\right)=-\sum_{A\subseteq X}\frac{\left|A\right|m_{2}\left(A\right)}{\left|X\right|}\log_{2}\frac{m_{2}\left(A\right)}{2^{\left|A\right|}-1}=0.1563,$$

$$E_{Wd}\left(m_{3}\right)=-\sum_{A\subseteq X}\frac{\left|A\right|m_{3}\left(A\right)}{\left|X\right|}\log_{2}\frac{m_{3}\left(A\right)}{2^{\left|A\right|}-1}=0.9647,$$

$$E_{Wd}\left(m_{4}\right)=-\sum_{A\subseteq X}\frac{\left|A\right|m_{4}\left(A\right)}{\left|X\right|}\log_{2}\frac{m_{4}\left(A\right)}{2^{\left|A\right|}-1}=0.9921,$$

$$E_{Wd}\left(m_{5}\right)=-\sum_{A\subseteq X}\frac{\left|A\right|m_{5}\left(A\right)}{\left|X\right|}\log_{2}\frac{m_{5}\left(A\right)}{2^{\left|A\right|}-1}=0.9225.$$

For the 3rd step, the weight of each evidence (BOE) can be calculated with Equation (18), the calculation results are as follows:

$$w_{1}=\frac{E_{Wd}\left(m_{1}\right)}{\sum_{i=1}^{5}E_{Wd}\left(m_{i}\right)}=0.1468,$$

$$w_{2}=\frac{E_{Wd}\left(m_{2}\right)}{\sum_{i=1}^{5}E_{Wd}\left(m_{i}\right)}=0.0439,$$

$$w_{3}=\frac{E_{Wd}\left(m_{3}\right)}{\sum_{i=1}^{5}E_{Wd}\left(m_{i}\right)}=0.2712,$$

$$w_{4}=\frac{E_{Wd}\left(m_{4}\right)}{\sum_{i=1}^{5}E_{Wd}\left(m_{i}\right)}=0.2789,$$

$$w_{5}=\frac{E_{Wd}\left(m_{5}\right)}{\sum_{i=1}^{5}E_{Wd}\left(m_{i}\right)}=0.2593.$$

For the 4th step, with Equation (19), the weighted mass function of each proposition in Table 2 is calculated as follows:

$$m_{w}\left(A\right)=\sum_{i=1}^{5}w_{i}m_{i}\left(A\right)=0.5264,$$

$$m_{w}\left(B\right)=\sum_{i=1}^{5}w_{i}m_{i}\left(B\right)=0.1549,$$

$$m_{w}\left(C\right)=\sum_{i=1}^{5}w_{i}m_{i}\left(C\right)=0.0484,$$

$$m_{w}\left(A,C\right)=\sum_{i=1}^{5}w_{i}m_{i}\left(A,C\right)=0.2703.$$

Finally, for the 5th step, with Dempster’s rule of combination and Equation (20), each of the new weighted mass function is fused four times. The fusion results are shown as follows:

$$m\left(A\right)={\left({\left({\left({\left(m_{w}⊕m_{w}\right)}_{1}⊕m_{w}\right)}_{2}⊕m_{w}\right)}_{3}⊕m_{w}\right)}_{4}\left(A\right)=0.9895,$$

$$m\left(B\right)={\left({\left({\left({\left(m_{w}⊕m_{w}\right)}_{1}⊕m_{w}\right)}_{2}⊕m_{w}\right)}_{3}⊕m_{w}\right)}_{4}\left(B\right)=0.0003,$$

$$m\left(C\right)={\left({\left({\left({\left(m_{w}⊕m_{w}\right)}_{1}⊕m_{w}\right)}_{2}⊕m_{w}\right)}_{3}⊕m_{w}\right)}_{4}\left(C\right)=0.0057,$$

$$m\left(A,C\right)={\left({\left({\left({\left(m_{w}⊕m_{w}\right)}_{1}⊕m_{w}\right)}_{2}⊕m_{w}\right)}_{3}⊕m_{w}\right)}_{4}\left(A,C\right)=0.0045.$$

With the proposed method, it can be concluded that target *A* is the recognized target. The results of this experiment with different methods are shown in Table 3. Although the experiment results with the methods in [11,58,59] all get a high belief on target *A*, the proposed method has the highest belief (98.95%) on the recognized target *A*. In addition, in [11], the method for evidence modification is based on evidence distance and Deng entropy simultaneously, which is not convincing, because both evidence distance and Deng entropy are based on mass functions of BOE, thus there exists a coupling relationship among those two indices in [11]. Compared with the methods in [11,58,59], the weighted belief entropy in the new method contributes to a stronger capacity in conflict data fusion by addressing more available uncertain information in BOE.

## 6. Application in Fault Diagnosis

In this section, the proposed method is applied to an experiment of fault diagnosis for a motor rotor. The practical data in [16] is adopted for the convenience of making a comparative study with some other methods.

### 6.1. Problem Description

According to [16], suppose there are three types of fault in a motor rotor, denoted as $F_{1}=\left\{Rotor\;\;unbalance\right\}$, $F_{2}=\left\{Rotor\;\;misalignment\right\}$ and $F_{3}=\left\{Pedestal\;\;looseness\right\}$, respectively. Three vibration acceleration sensors are placed in different installation positions to collect the vibration signal. The acceleration vibration frequency amplitudes at the frequencies of Freq1, Freq2 and Freq3 are taken as the fault feature variables. The results of sensor reports modelled as BOEs are presented in Table 4, where $m_{s_{1}}\left(\cdot \right)$, $m_{s_{2}}\left(\cdot \right)$ and $m_{s_{3}}\left(\cdot \right)$ denote the BOEs reported from these three vibration acceleration sensors.

### 6.2. Data Fusion Based on the New Method

Execute the method presented in Section 4 to solve the fault diagnosis problem mentioned above.

**Step 1** Uncertain data modeling with BPA.

In this paper, BPAs of sensor reports are directly adopted from [16], as is shown in Table 4. In real applications, how to model uncertain information with BPAs is an open issue [60,61], which is not the scope of this paper. For more information about generating BPAs of Table 4, please refer to [16].

**Step 2** Uncertainty measure of BPA with weighted belief entropy.

In the proposed method, the uncertainty of sensor reports is measured based on the weighted belief entropy. With Equation (17), the weighted belief entropy of each BOE under the vibration acceleration frequency of Freq1 is calculated as follows:

$$E_{Wd}\left(m_{s_{1}}\right)=-\sum_{A\subseteq X}\frac{\left|A\right|m_{s_{1}}\left(A\right)}{\left|X\right|}\log_{2}\frac{m_{s_{1}}\left(A\right)}{2^{\left|A\right|}-1}=0.5657,$$

$$E_{Wd}\left(m_{s_{2}}\right)=-\sum_{A\subseteq X}\frac{\left|A\right|m_{s_{2}}\left(A\right)}{\left|X\right|}\log_{2}\frac{m_{s_{2}}\left(A\right)}{2^{\left|A\right|}-1}=0.7096,$$

$$E_{Wd}\left(m_{s_{3}}\right)=-\sum_{A\subseteq X}\frac{\left|A\right|m_{s_{3}}\left(A\right)}{\left|X\right|}\log_{2}\frac{m_{s_{3}}\left(A\right)}{2^{\left|A\right|}-1}=0.7206.$$

Similarly, the weighted belief entropy of sensor reports under Freq2 and Freq3 can be calculated, and the results are shown in Table 5.

**Step 3** Calculate the weight of each BOE.

With Equation (18), for the vibration acceleration frequency of Freq1, the weight of each BOE for evidence modification is calculated as follows:

$$w_{S_{1}}=\frac{E_{Wd}\left(m_{s_{1}}\right)}{\sum_{i=1}^{3}E_{Wd}\left(m_{s_{i}}\right)}=\frac{0.5657}{0.5657+0.7096+0.7206}=0.2834,$$

$$w_{S_{2}}=\frac{E_{Wd}\left(m_{s_{2}}\right)}{\sum_{i=1}^{3}E_{Wd}\left(m_{s_{i}}\right)}=\frac{0.7096}{0.5657+0.7096+0.7206}=0.3555,$$

$$w_{S_{3}}=\frac{E_{Wd}\left(m_{s_{3}}\right)}{\sum_{i=1}^{3}E_{Wd}\left(m_{s_{i}}\right)}=\frac{0.7206}{0.5657+0.7096+0.7206}=0.3610.$$

The weight of different sensor reports under Freq2 and Freq3 is shown in Table 6.

**Step 4** Calculate the weighted mass functions.

With Equation (19), the modified mass function for each judgement on fault types with respect to Freq1 can be calculated as follows:

$m_{w}\left(\left\{F2\right\}\right)=\sum_{i=1}^{3}w_{s_{i}}m_{i}\left(\left\{F2\right\}\right)=0.2834\times 0.8176+0.3555\times 0.5658+0.3610\times 0.2403=0.5196,$
$m_{w}\left(\left\{F3\right\}\right)=\sum_{i=1}^{3}w_{s_{i}}m_{i}\left(\left\{F3\right\}\right)=0.2834\times 0.0003+0.3555\times 0.0009+0.3610\times 0.0004=0.0006,$
$m_{w}\left(\left\{F1,F2\right\}\right)=\sum_{i=1}^{3}w_{s_{i}}m_{i}\left(\left\{F1,F2\right\}\right)=0.2834\times 0.1553+0.3555\times 0.0646+0.3610\times 0.0141=\;0.0721,$
$m_{w}\left(\left\{F1,F2,F3\right\}\right)=\sum_{i=1}^{3}w_{s_{i}}m_{i}\left(\left\{F1,F2,F3\right\}\right)=0.2834\times 0.0268+0.3555\times 0.3687+0.3610\times 0.7452=0.4077.$

The modified mass function for Freq2 and Freq3 can also be calculated with Equation (19), and the results are shown in Table 7.

**Step 5** Data fusion with Dempster’s rule of combination.

With Equation (20), for the vibration acceleration frequency of Freq1, the modified mass function will be fused with Dempster’s rule of combination two times, shown as follows:

$$m\left(\left\{F2\right\}\right)={\left({\left(m_{w}⊕m_{w}\right)}_{1}⊕m_{w}\right)}_{2}\left(\left\{F2\right\}\right)=0.8891,$$

$$m\left(\left\{F3\right\}\right)={\left({\left(m_{w}⊕m_{w}\right)}_{1}⊕m_{w}\right)}_{2}\left(\left\{F3\right\}\right)=0.0003,$$

$$m\left(\left\{F1,F2\right\}\right)={\left({\left(m_{w}⊕m_{w}\right)}_{1}⊕m_{w}\right)}_{2}\left(\left\{F1,F2\right\}\right)=0.0427,$$

$$m\left(\left\{F1,F2,F3\right\}\right)={\left({\left(m_{w}⊕m_{w}\right)}_{1}⊕m_{w}\right)}_{2}\left(\left\{F1,F2,F3\right\}\right)=0.0679.$$

The fusion results for Freq2 and Freq3 are shown in Table 8.

### 6.3. Discussion

The result of fault diagnosis, according to Table 8, is that F2 is the fault type. The conflict of sensor reports in the problem, e.g., under Freq2, the belief on F2 is 0.8176, 0.5658 and 0.2403, respectively, is overcome with the new method. According to Table 9, the fusion result is consistent with the method in [16]. In addition, the fusion result with the proposed method has a higher support degree on the decision that F2 is the diagnosis result in comparison with the method in [16].

Three reasons contribute to the effectiveness and superiority of the new multi-sensor data fusion method. Firstly, the new method is based on the new uncertainty measure, the new measure can address more uncertain information in the Dempster–Shafer evidence theory framework, which contributes to a more accurate experiment result in comparison with [16]. Secondly, the sensor data is preprocessed properly with the new uncertainty measure in the proposed sensor data fusion method, which is very important in combining conflict evidence. Finally, the merits of Dempster’s rule, such as satisfying the rule of commutativity and associativity, guarantee the rationality of the fusion result.

## 7. Conclusions

In this paper, in the Dempster–Shafer evidence theory framework, the weighted belief entropy is proposed based on Deng entropy. The new measure takes advantage of information included in, not only the mass function, but also the scale of the FOD. By addressing more information in a BOE, which means less information loss in information processing, the weighted belief entropy can quantify the uncertainty of evidence effectively. The numerical example shows that this new measure can quantify the uncertainty of evidence more accurately, which is helpful for information processing.

Based on the weighted belief entropy, a multi-sensor data fusion approach is proposed in this paper. A numerical example and an application on fault diagnosis are presented to verify the rationality and effectiveness of the new sensor data fusion method. Both the numerical example and the application indicate that the new measure contributes to a more accurate sensor data fusion method by addressing more uncertain information in the Dempster–Shafer evidence theory framework (BOE).

Further study of this work will be focused on extending the new measure and the proposed multi-sensor data fusion approach to solve more problems in industrial applications.

## Figures and Tables

**Table 1:** Comparison between the weighted belief entropy and Deng entropy with a variable proposition *T*.

Cases	Deng Entropy	Weighted Belief Entropy
$T=\left\{1\right\}$	2.6623	2.5180
$T=\left\{1,2\right\}$	3.9303	3.7090
$T=\left\{1,2,3\right\}$	4.9082	4.6100
$T=\left\{1,...,4\right\}$	5.7878	5.4127
$T=\left\{1,...,5\right\}$	6.6256	6.1736
$T=\left\{1,...,6\right\}$	7.4441	6.9151
$T=\left\{1,...,7\right\}$	8.2532	7.6473
$T=\left\{1,...,8\right\}$	9.0578	8.3749
$T=\left\{1,...,9\right\}$	9.8600	9.1002
$T=\left\{1,...,10\right\}$	10.6612	9.8244
$T=\left\{1,...,11\right\}$	11.4617	10.5480
$T=\left\{1,...,12\right\}$	12.2620	11.2714
$T=\left\{1,...,13\right\}$	13.0622	11.9946
$T=\left\{1,...,14\right\}$	13.8622	12.7177

**Table 2:** Basic probability assignment (BPA) of artificial data.

BPA	m(A)	m(B)	m(C)	m(A,C)
1st Sensor report: $m_{1}\left(\cdot \right)$	0.41	0.29	0.3	0
2nd Sensor report: $m_{2}\left(\cdot \right)$	0	0.9	0.1	0
3rd Sensor report: $m_{3}\left(\cdot \right)$	0.58	0.07	0	0.35
4th Sensor report: $m_{4}\left(\cdot \right)$	0.55	0.1	0	0.35
5th Sensor report: $m_{5}\left(\cdot \right)$	0.6	0.1	0	0.3

**Table 3:** Experimental results with different methods.

Methods	m(A)	m(B)	m(C)	m(A,C)
Deng et al.’s method [58]	0.9820	0.0039	0.0107	0.0034
Zhang et al.’s method [59]	0.9820	0.0033	0.0115	0.0032
Yuan et al.’s method [11]	0.9886	0.0002	0.0072	0.0039
The proposed method	0.9895	0.0003	0.0057	0.0045

**Table 4:** Data for fault diagnosis modelled as BPAs [16].

			*Freq*1				*Freq*2				*Freq*3	
	{F2}	{F3}	{F1,F2}	{F1,F2,F3}		{F2}	{F1,F2,F3}		{F1}	{F2}	{F1,F2}	{F1,F2,F3}
$m_{s_{1}}\left(\cdot \right)$	0.8176	0.0003	0.1553	0.0268		0.6229	0.3771		0.3666	0.4563	0.1185	0.0586
$m_{s_{2}}\left(\cdot \right)$	0.5658	0.0009	0.0646	0.3687		0.7660	0.2341		0.2793	0.4151	0.2652	0.0404
$m_{s_{3}}\left(\cdot \right)$	0.2403	0.0004	0.0141	0.7452		0.8598	0.1402		0.2897	0.4331	0.2470	0.0302

**Table 5:** Weighted belief entropy of sensor reports under different frequencies.

$E_{Wd}\left(\cdot \right)$	Freq1	Freq2	Freq3
$E_{Wd}\left(m_{s_{1}}\right)$	0.5657	0.4596	0.7983
$E_{Wd}\left(m_{s_{2}}\right)$	0.7096	0.3277	1.0257
$E_{Wd}\left(m_{s_{3}}\right)$	0.7206	0.2207	0.9875

**Table 6:** Weighted belief entropy of sensor reports under different frequencies.

$w_{S_{i}}$	Freq1	Freq2	Freq3
$w_{S_{1}}$	0.2834	0.4560	0.2839
$w_{S_{2}}$	0.3555	0.3251	0.3648
$w_{S_{3}}$	0.3610	0.2189	0.3513

**Table 7:** Modified mass function.

			Freq1				Freq2				Freq3	
	{F2}	{F3}	{F1,F2}	{F1,F2,F3}		{F2}	{F1,F2,F3}		{F1}	{F2}	{F1,F2}	{F1,F2,F3}
$m_{w}\left(\cdot \right)$	0.5196	0.0006	0.0721	0.4077		0.7212	0.2788		0.3077	0.4331	0.2172	0.0420

**Table 8:** Sensor data fusion results for fault diagnosis.

			Freq1				Freq2				Freq3	
	{F2}	{F3}	{F1,F2}	{F1,F2,F3}		{F2}	{F1,F2,F3}		{F1}	{F2}	{F1,F2}	{F1,F2,F3}
Fusion result	0.8891	0.0003	0.0427	0.0679		0.9784	0.0216		0.3318	0.6332	0.0349	0.0001

**Table 9:** Comparison of results obtained using proposed method and Jiang et al. method.

Method			Freq1				Freq2				Freq3	
	{F2}	{F3}	{F1,F2}	{F1,F2,F3}		{F2}	{F1,F2,F3}		{F1}	{F2}	{F1,F2}	{F1,F2,F3}
Jiang et al.’s method [16]	**0.8861**	0.0002	0.0582	0.0555		**0.9621**	0.0371		0.3384	**0.5904**	0.0651	0.0061
Proposed method	**0.8891**	0.0003	0.0427	0.0679		**0.9784**	0.0216		0.3318	**0.6332**	0.0349	0.0001

## References

[1] Alexandridis A. (2013). Evolving RBF neural networks for adaptive soft-sensor design. Int. J. Neural Syst..

[2] Marinkovic Z., Atanaskovic A., Xibilia M.G., Pace C., Latino M., Donato N. A neural network approach for safety monitoring applications. Proceedings of the 2016 IEEE Sensors Applications Symposium (SAS).

[3] Graziani S., Pagano F., Xibilia M.G. Soft sensor for a propylene splitter with seasonal variations. Proceedings of the 2010 IEEE Instrumentation Measurement Technology Conference (I2MTC).

[4] Caponetto R., Dongola G., Gallo A., Xibilia M.G. FPGA Implementation of a soft sensor for the estimation of the freezing point of kerosene. Proceedings of the ASME 2009 International Design Engineering Technical Conferences and Computers and Information in Engineering Conference.

[5] Geng H., Liang Y., Yang F., Xu L., Pan Q. (2016). Model-reduced fault detection for multi-rate sensor fusion with unknown inputs. Inf. Fusion.

[6] Reiche J., Verbesselt J., Hoekman D., Herold M. (2015). Fusing Landsat and SAR time series to detect deforestation in the tropics. Remote Sens. Environ..

[7] Ma J., Zhao J., Ma Y., Tian J. (2015). Non-rigid visible and infrared face registration via regularized Gaussian fields criterion. Pattern Recognit..

[8] Jiang D., Zhuang D., Huang Y., Fu J. (2009). Advances in multi-sensor data fusion: Algorithms and applications. Sensors.

[9] Fortuna L., Graziani S., Xibilia M.G. (2009). Comparison of soft-sensor design methods for industrial plants using small data sets. IEEE Trans. Instrum. Meas..

[10] Frikha A., Moalla H. (2015). Analytic hierarchy process for multi-sensor data fusion based on belief function theory. Eur. J. Oper. Res..

[11] Yuan K., Xiao F., Fei L., Kang B., Deng Y. (2016). Conflict management based on belief function entropy in sensor fusion. SpringerPlus.

[12] Jiang W., Wei B., Xie C., Zhou D. (2016). An evidential sensor fusion method in fault diagnosis. Adv. Mech. Eng..

[13] Yuan K., Xiao F., Fei L., Kang B., Deng Y. (2016). Modeling sensor reliability in fault diagnosis based on evidence Theory. Sensors.

[14] Jiang W., Wei B., Qin X., Zhan J., Tang Y. (2016). Sensor data fusion based on a new conflict measure. Math. Probl. Eng..

[15] Chen S., Deng Y., Wu J. (2013). Fuzzy sensor fusion based on evidence theory and its application. Appl. Artif. Intell..

[16] Jiang W., Xie C., Zhuang M., Shou Y., Tang Y. (2016). Sensor data fusion with z-numbers and its application in fault diagnosis. Sensors.

[17] Gao C., Yan C., Adamatzky A., Deng Y. (2014). A bio-inspired algorithm for route selection in wireless sensor networks. IEEE Commun. Lett..

[18] Dempster A.P. (1967). Upper and lower probabilities induced by a multi-valued mapping. Ann. Math. Stat..

[19] Shafer G. (1976). A Mathematical Theory of Evidence.

[20] Chin K.S., Fu C., Wang Y. (2015). A method of determining attribute weights in evidential reasoning approach based on incompatibility among attributes. Comput. Ind. Eng..

[21] Du W.S., Hu B.Q. (2016). Attribute reduction in ordered decision tables via evidence theory. Inf. Sci..

[22] Fu C., Wang Y. (2015). An interval difference based evidential reasoning approach with unknown attribute weights and utilities of assessment grades. Comput. Ind. Eng..

[23] Wang Y.M., Elhag T.M.S. (2007). A comparison of neural network, evidential reasoning and multiple regression analysis in modelling bridge risks. Expert Syst. Appl..

[24] Su X., Deng Y., Mahadevan S., Bao Q. (2012). An improved method for risk evaluation in failure modes and effects analysis of aircraft engine rotor blades. Eng. Fail. Anal..

[25] Fu C., Yang J.B., Yang S.L. (2015). A group evidential reasoning approach based on expert reliability. Eur. J. Oper. Res..

[26] Zhang X., Mahadevan S., Deng X. (2017). Reliability analysis with linguistic data: An evidential network approach. Reliab. Eng. Syst. Saf..

[27] Jiang W., Xie C., Wei B., Zhou D. (2016). A modified method for risk evaluation in failure modes and effects analysis of aircraft turbine rotor blades. Adv. Mech. Eng..

[28] Su X., Mahadevan S., Xu P., Deng Y. (2015). Dependence assessment in human reliability analysis using evidence theory and AHP. Risk Anal..

[29] Denoeux T. (1995). A k-nearest neighbor classification rule based on Dempster–Shafer theory. IEEE Trans. Syst. Man Cybern..

[30] Liu Z.G., Pan Q., Dezert J. (2013). A new belief-based K-nearest neighbor classification method. Pattern Recognit..

[31] Ma J., Liu W., Miller P., Zhou H. (2015). An evidential fusion approach for gender profiling. Inf. Sci..

[32] Liu Z.G., Pan Q., Dezert J., Mercier G. (2014). Credal classification rule for uncertain data based on belief functions. Pattern Recognit..

[33] Han D., Liu W., Dezert J., Yang Y. (2016). A novel approach to pre-extracting support vectors based on the theory of belief functions. Knowl.-Based Syst..

[34] Liu Z.G., Pan Q., Dezert J., Martin A. (2016). Adaptive imputation of missing values for incomplete pattern classification. Pattern Recognit..

[35] Yager R.R., Filev D.P. (1995). Including probabilistic uncertainty in fuzzy logic controller modeling using Dempster–Shafer theory. IEEE Trans. Syst. Man Cybern..

[36] Tang Y., Zhou D., Jiang W. (2016). A new fuzzy-evidential controller for stabilization of the planar inverted pendulum system. PLoS ONE.

[37] Wang Y.M., Yang J.B., Xu D.L., Chin K.S. (2009). Consumer preference prediction by using a hybrid evidential reasoning and belief rule-based methodology. Expert Syst. Appl..

[38] Ma J., Liu W., Benferhat S. (2015). A belief revision framework for revising epistemic states with partial epistemic states. Int. J. Approx. Reason..

[39] Zhou K., Martin A., Pan Q., Liu Z.G. (2015). Median evidential c-means algorithm and its application to community detection. Knowl.-Based Syst..

[40] Zadeh L.A. (1986). A simple view of the Dempster–Shafer theory of evidence and its implication for the rule of combination. AI Mag..

[41] Deng Y. (2015). Generalized evidence theory. Appl. Intell..

[42] Fan X., Zuo M.J. (2006). Fault diagnosis of machines based on D-S evidence theory. Part 1: D–S evidence theory and its improvement. Pattern Recognit. Lett..

[43] Yang Y., Han D. (2016). A new distance-based total uncertainty measure in the theory of belief functions. Knowl.-Based Syst..

[44] Song Y., Wang X., Lei L., Yue S. (2015). Uncertainty measure for interval-valued belief structures. Measurement.

[45] Song Y., Wang X., Zhang H. (2015). A distance measure between intuitionistic fuzzy belief functions. Knowl.-Based Syst..

[46] Shannon C.E. (2001). A mathematical theory of communication. ACM SIGMOBILE Mob. Comput. Commun. Rev..

[47] Chen Z., Dehmer M., Shi Y. (2014). A note on distance-based graph entropies. Entropy.

[48] Cao S., Dehmer M., Shi Y. (2014). Extremality of degree-based graph entropies. Inf. Sci..

[49] Chen Z., Dehmer M., Emmert-Streib F., Shi Y. (2014). Entropy bounds for dendrimers. Appl. Math. Comput..

[50] Cao S., Dehmer M. (2015). Degree-based entropies of networks revisited. Appl. Math. Comput..

[51] Hohle U. Entropy with respect to plausibility measures. Proceedings of the Proceedings of the 12th IEEE International Symposium on Multiple-Valued Logic.

[52] Yager R.R. (1983). Entropy and specificity in a mathematical theory of evidence. Int. J. Gen. Syst..

[53] Dubois D., Prade H. (1985). A note on measures of specificity for fuzzy sets. Int. J. Gen. Syst..

[54] Klir G.J., Ramer A. (1991). Uncertainty in Dempster–Shafer theory: A critical re-examination. Int. J. Gen. Syst..

[55] Klir G.J., Parviz B. A note on the measure of discord. Proceedings of the Eighth International Conference on Uncertainty in Artificial Intelligence.

[56] George T., Pal N.R. (1996). Quantification of conflict in Dempster-Shafer framework: A new approach. Int. J. Gen. Syst..

[57] Deng Y. (2016). Deng entropy. Chaos Solitons Fractals.

[58] Deng Y., Shi W., Zhu Z., Liu Q. (2004). Combining belief functions based on distance of evidence. Decis. Support Syst..

[59] Zhang Z., Liu T., Chen D., Zhang W. (2014). Novel algorithm for identifying and fusing conflicting data in wireless sensor networks. Sensors.

[60] Deng X., Liu Q., Deng Y., Mahadevan S. (2016). An improved method to construct basic probability assignment based on the confusion matrix for classification problem. Inf. Sci..

[61] Jiang W., Zhan J., Zhou D., Li X. (2016). A method to determine generalized basic probability assignment in the open world. Math. Probl. Eng..

